# Dissecting the functional anatomy of auditory word repetition

**DOI:** 10.3389/fnhum.2014.00246

**Published:** 2014-05-06

**Authors:** Thomas M. H. Hope, Susan Prejawa, ‘Ōiwi Parker Jones, Marion Oberhuber, Mohamed L. Seghier, David W. Green, Cathy J. Price

**Affiliations:** ^1^Wellcome Trust Centre for Neuroimaging, Institute of Neurology, University College LondonLondon, UK; ^2^Wolfson College, University of OxfordOxford, UK; ^3^Department of Cognitive, Perceptual and Brain Sciences, University College LondonLondon, UK

**Keywords:** fMRI, language, auditory word repetition

## Abstract

This fMRI study used a single, multi-factorial, within-subjects design to dissociate multiple linguistic and non-linguistic processing areas that are all involved in repeating back heard words. The study compared: (1) auditory to visual inputs; (2) phonological to non-phonological inputs; (3) semantic to non-semantic inputs; and (4) speech production to finger-press responses. The stimuli included words (semantic and phonological inputs), pseudowords (phonological input), pictures and sounds of animals or objects (semantic input), and colored patterns and hums (non-semantic and non-phonological). The speech production tasks involved auditory repetition, reading, and naming while the finger press tasks involved one-back matching. The results from the main effects and interactions were compared to predictions from a previously reported functional anatomical model of language based on a meta-analysis of many different neuroimaging experiments. Although many findings from the current experiment replicated many of those predicted, our within-subject design also revealed novel results by providing sufficient anatomical precision to dissect several different regions within the anterior insula, pars orbitalis, anterior cingulate, SMA, and cerebellum. For example, we found one part of the pars orbitalis was involved in phonological processing and another in semantic processing. We also dissociated four different types of phonological effects in the left superior temporal sulcus (STS), left putamen, left ventral premotor cortex, and left pars orbitalis. Our findings challenge some of the commonly-held opinions on the functional anatomy of language, and resolve some previously conflicting findings about specific brain regions—and our experimental design reveals details of the word repetition process that are not well captured by current models.

## Introduction

Although auditory word repetition is amongst the simplest of language tasks, it involves many different brain regions whose functions are not yet fully understood. The aim of this paper was to dissociate the brain regions that support 10 different levels of processing, which are all thought to occur during auditory repetition. Importantly, all 10 levels of processing were investigated using a within-subject, fully-balanced factorial design that enables functional anatomy to be dissected at a spatial precision beyond that possible when results are compiled from multiple studies, conducted on different participant samples.

We start by (A) considering the sensorimotor and cognitive functions involved in auditory word repetition. We then (B) describe how our within-subjects experimental design is able to dissociate the brain areas supporting 10 different functions and (C) make predictions of the brain areas associated with each function based on hundreds of prior neuroimaging studies that each investigated only a small subset of the functions reported in the current study.

(A) *Functional models of auditory word repetition*.

Auditory word repetition requires the immediate reproduction of a word that has been spoken by someone else. In essence, it involves translating an auditory input into articulatory activity (i.e., the mouth movements, breathing, and laryngeal activity) that is required to produce an auditory output that matches the identity of the heard word. In most cognitive models, the mapping of auditory inputs to articulatory activity is mediated by previously-learnt representations of speech sounds (phonology), with further support from the semantic system when the speech has meaning (Hanley et al., 2002, 2004).

Standard cognitive models of speech and reading make a distinction between input phonology and output phonology (e.g., Patterson and Shewell, 1987; Ellis and Young, 1988; see Harley, 2001 for a review). Input phonology supports speech perception, when auditory speech inputs are linked to prior knowledge of speech sounds. Output phonology supports speech production when prior knowledge of speech sounds drives and monitors articulatory activity (Tourville and Guenther, 2011; Guenther and Vladusich, 2012). Neuroimaging studies have also distinguished an intermediate level of phonological processing that is actively involved in recoding auditory speech inputs into vocal tract gestures (Zatorre et al., 1992; Hickok et al., 2003). This is referred to as “articulatory recoding” or “sensori-motor integration.”

**Figure 1:**
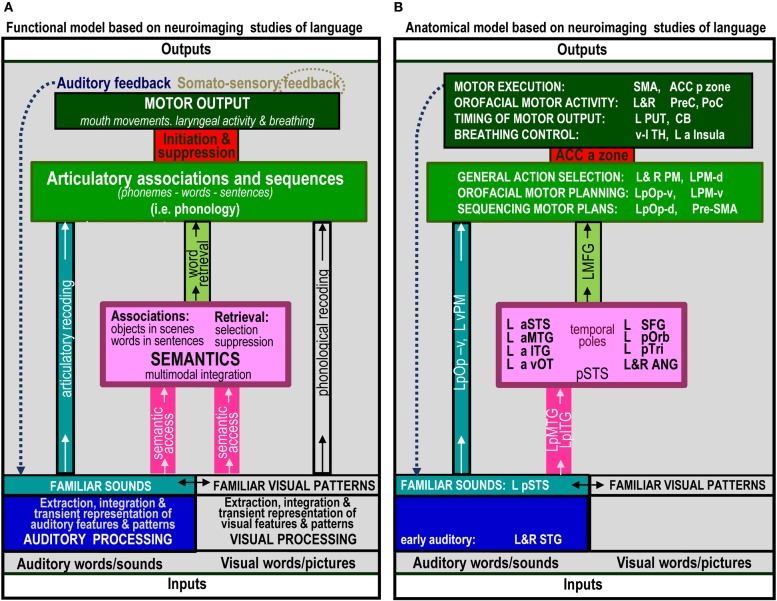
**The functional anatomy of auditory word repetition, based on an anatomical model of language in Price (2012) inspired by a review of hundreds of fMRI experiments**. Parts that are not related to processes tested in the current experiment have been excluded. **(A)** defines the processing functions. **(B)** lists the brain areas that were associated with each function in Price (2012). Abbreviated names are explained in Table 4.

The range of auditory repetition processes that we investigated in this study was determined by two considerations: (1) a priori predictions based on a single functional anatomical model of language that emerged from a review of 20 years of functional neuroimaging studies in healthy participants (Price, 2012); and (2) the limits of a single, within-subjects fMRI design. Figure 1 illustrates the components of the functional-anatomical model of language reported in Price (2012) after removing the components that are not directly related to auditory word repetition or our experimental design. Our analysis focuses on 10 processes, extracted from this model. These are listed and described in Table 1A, for easy reference when describing the statistical contrasts (Table 1B), predictions (Table 2A), and results (Table 2B).

**Table 1:**
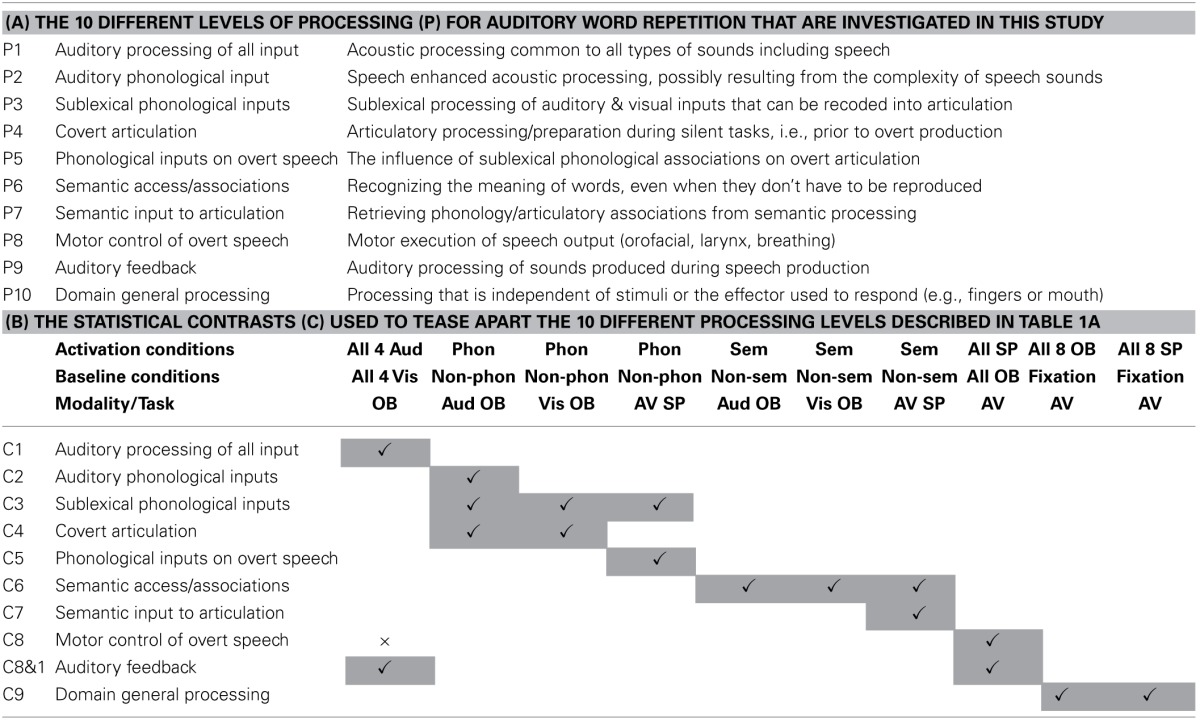
**(A) Task decomposition for auditory word repetition into 10 processing functions of interest (P1–P10); (B) The 10 statistical contrasts (C1–C9, with two variants of C8) used to identify regional responses to each of the 10 processing functions of interest in (A)**.

*The dark boxes with ticks indicate the column that shows the activation condition (top row) and baseline condition (second row) used in the statistical contrast. The third row indicates the stimulus modality or task that was kept constant across the activation and baseline condition. Contrast 8 (SP > OB) is repeated twice, once with an additional tick in the first column (AUD-VIS OB) and once with a white box and cross. This was to dissociate speech production areas into those that were (A) in auditory processing areas that were likely to be associated with auditory processing in response to the participants' own voices; and (B) not in auditory processing areas and therefore more likely to be related to the motor execution of speech*.

Abbreviations: Phon, Phonological inputs (words and pseudowords); Non-phon, Nonphonological inputs; Sem, Semantic inputs (words, pictures & environmental sounds); Non-sem, Non-semantic inputs; OB, One back matching task; SP, Speech production task; Both, SP and OB; Aud, Auditory stimuli; Vis, Visual stimuli; AV, Auditory & visual stimuli

**Table 2:** **(A) Brain areas that were predicted, a priori, for each of the 10 processing functions of interest (P1–P10) according to an extensive review of the literature (see Table 2 in Price, 2012). (B) Brain areas that were identified for each of the 10 statistical contrasts of interest (C1–C9, with two variants of C8)**.

**(A) ANATOMICAL LOCATION OF PREDICTED ACTIVATIONS FOR P1–10 IN TABLE 1A. PREDICTIONS FROM TABLE 2 in PRICE (2012)**		
P1	Auditory processing of all input	Bilateral STG, including Heschl's gyri & plana temporale
P2	Auditory phonological inputs	*Nothing predicted*
P3	Sublexical phonological inputs	Left pSTS (representations of familiar sounds); v-pOp, v-pM (articulatory recoding)
P4	Covert articulation	Left v-pOp, d-pOp, bilateal PM, pre-SMA
P5	Phonological inputs on overt speech	Not predicted but could increase demands on articulatory associations (P4) or motor responses (P8–10)
P6	Semantic access/associations	Left pMTG & pITG
P7	Semantic input to articulation	Left MFG
P8	Motor control of overt speech	PreC, poC, CB (IV, V, VI, VIII), a-INS, PUT, thalamus
P9	Auditory feedback	As for P1
P10	Domain general processing	ACC, SMA, pre-SMA, Left d-pOp/d-PM
**(B) RESULTS THAT WERE INCONSISTENT WITH PREDICTIONS IN TABLE 2A ARE BOLD. SEE TEXT FOR DETAILS AND TABLE 4**		
**FOR ABBREVIATIONS**		
C1	Auditory processing of all input	*As predicted*
C2	Auditory phonological inputs	*As predicted*
C3	Sublexical phonological inputs	Left pSTS, **left aSTs, left PUT-p** (not v-pOp, v-pM)
C4	Covert articulation	**Left pOrb/pTri** (Not pOp, PM, pre-SMA)
C5	Phonological inputs on overt speech	**Left v-PM & Left PUT-a**
C6	Semantic access/associations	**Left pOrb** (not MTG or ITG)
C7	Semantic input to articulation	**Left p-MTG, ANG, hippocampus, right CB (VI crux 1; VIIB), left FO, pOrb, pTri, IFS** (not MFG)
C8	Motor control of overt speech	preC, poC, CB (V,VI, VIII), a-INS, PUT-v, **amygdala, Tp, pTri, SMA, ACC** (not thalamus)
C8&1	Auditory feedback	*As predicted*
C9	Domain general processing	ACC, SMA, pre-SMA, Left d-pOp/d-PM, **vPM, aINS-d, SMG, lateral CB**

*Highlighted in bold font are those that were inconsistent with the predictions in Table 2A above. Regions in parentheses were predicted to respond, but did not. See Table 4 for region name abbreviations*.

The 10 processing functions of interest were (P1) auditory processing of familiar and unfamiliar stimuli; (P2) recognition of familiar speech sounds, i.e., auditory phonological input processing; (P3) access to sublexical representations of speech sounds that can be recoded / integrated with articulatory associations; (P4) covert articulatory processing; (P5) sublexical phonological processing of words and pseudowords that influences the motor execution of speech, for example because sublexical phonological cues increase the demands on articulation sequences; (P6) accessing semantic knowledge; (P7) retrieving articulatory plans required to produce words from semantic concepts, as opposed to phonological clues; (P8) motor execution of speech output including orofacial and larynx activity, breathing and the timing of response; (P9) auditory processing of the sound of the spoken response; and (P10) domain general processing that occurs for all types of stimuli and response.

(B) *Our within-subjects fMRI design for teasing apart multiple processing areas*.

To tease apart brain regions that are involved in different processes or functions underlying auditory repetition, we used fMRI to compare brain activation during auditory repetition to brain activation during tasks that each activate a subset of the functions of interest. Altogether, there were four different experimental factors: (1) stimulus modality: auditory vs. visual stimuli; (2) sublexical phonological input: phonological vs. non-phonological stimuli; (3) semantic content: semantic vs. non-semantic stimuli; and (4) speech production: speech production vs. a one-back matching task (with finger press response). This resulted in 16 different conditions (i.e., 2 × 2 × 2 × 2), including auditory word repetition (a task involving auditory stimuli, with phonological and semantic content, and requiring speech production in response). The other 15 conditions are listed in Table 3. With this design, we dissected the functions of those regions found to be active for auditory word repetition relative to fixation and then dissected these regions according to our 10 functions of interest (see Table 1 and Materials and Methods for further details). As with all experimental approaches, our rationale is based on our own assumptions about the level of processing that will be engaged in each condition of interest. The data allow us to test these assumptions by comparing the observed effects against those expected from previous studies.

**Table 3:** **A schematic representation of the 16 tasks employed in this work, associating each task with the key factors: stimulus modality (auditory vs. visual); process (semantic and/or phonological content); and response modality (SP vs. OB)**.

	**Speech production task (SP)**				**One back matching task (OB)**			
**Process**	**Semantics phonology**	**…… Phonology**	**Semantics ……**	**…… ……**	**Semantics phonology**	**…… Phonology**	**Semantics ……**	**…… ……**
**THE 16 DIFFERENT CONDITIONS USED TO TEASE APART DIFFERENT STAGES OF WORD REPETITION**								
Auditory	Word repetition	Pseudoword repetition	Sound naming	Gender naming	Word matching	Pseudoword matching	Sound matching	Gender matching
Visual	Word reading	Pseudoword reading	Picture naming	Color naming	Word matching	Pseudoword matching	Picture matching	Color matching

(C) *Predictions based on prior neuroimaging studies*.

In the last 20 years, there have been literally hundreds of studies that have used functional neuroimaging techniques, such as PET and fMRI, to reveal the brain areas that support different levels of language processing. The number of possible predictions for our 10 processing functions of interest therefore becomes unruly without constraints. To simplify the selection of predictions, we focus on the brain areas predicted by a single functional anatomical model of language based on a review of many hundreds of neuroimaging papers (Price, 2012). These predictions are provided in Figure 3 of Price (2012) with the anatomical components relevant to auditory word repetition shown in Figure 1B of the current paper (see Table 4 for the list of abbreviations). Those predictions specifically associated with our 10 processing functions of interest are listed in Table 2A. The results of each statistical contrast may then be considered according to whether or not they supported the predictions (see Table 2B). Our discussion focuses on novel findings that were not predicted *a priori*, and emphasizes the complexity of the brain networks that support auditory word repetition.

**Table 4:** **Abbreviations used in the text, tables, and figures**.

**A KEY TO THE ABBREVIATIONS**		
**ANATOMICAL NAME**		
ACC	Anterior cingulate gyrus	
ACC	Anterior cingulate sulcus	
aINS	Anterior Insula	
ANG	Angular gyrus	
CB	Cerebellum (lobule)	
CS	Central sulcus	
FO	Frontal operculum	
HG	Heschl's gyrus	
IFG	Inferior frontal gyrus	
IFS	Inferior frontal sulcus	
ITG	Inferior temporal gyrus	
MTG	Middle tempomral gyrus	
PM	Premotor cortex	
PoC	Postcentral	
PoCing	Posterior cingulate	
pOp	Pars opercularis (in IFG)	
pOrb	Pars orbitalis (in IFG)	
PrC	Precentral	PreC
PT	Planum temporale	
pTri	Pars triangularis (in IFG)	
PUT	Putamen	put
SFG	Superior frontal gyrus	
SMA	Supplementary motor area	
SMG	Supramarginal gyrus	
STG	Superior temporal gyrus	
STS	Superior temporal sulcus	
Tp	Temporal pole	
TPJ	Temporo-parietal-junction	
**QUALIFICATION ON LOCATION**		
a	Anterior zone	
d	Dorsal	
g	Gyrus/gyri	
H	Hemisphere	
L	Left hemisphere	
m	Medial	
p	Posterior zone	
R	Right hemisphere	
s	Sulcus	
v	Ventral	
C	Contrast (1–9), see Table 1B	

## Materials and methods

The study was approved by the London Queen Square Research Ethics Committee. All participants gave written informed consent prior to scanning and received financial compensation for their time.

### Participants

The participants were 25 healthy, right-handed, native speakers of English, with normal or corrected-to-normal vision; 12 females, 13 males, age range = 20–45 years, mean = 31.4 years, *SD* = 5.9 years. Handedness was assessed with the Edinburgh Handedness Inventory (Oldfield, 1971). A 26th participant was subsequently excluded from analyses because of data corruption in one condition.

### Experimental design

The condition of interest was auditory word repetition. This was embedded in a larger experimental design with a total of 16 different conditions, which allowed us to tease apart the brain regions supporting the sub-functions underlying auditory word repetition. The 16 conditions conformed to a 2 × 2 × 2 × 2 factorial design (see Table 3). In brief, Factor 1 was stimulus modality: auditory vs. visual modalities; Factor 2 was the presence or absence of sublexical phonological cues; Factor 3 was the presence or absence of familiar semantic content in the stimuli; and Factor 4 was speech production in response to the stimulus vs. one-back matching which involved a finger press response to indicate if the current stimulus was the same as the previous stimulus.

The stimuli with sublexical phonological cues and semantic content were auditory or visually presented words. The stimuli with sublexical phonological cues but no semantic content were auditory or visually presented pseudowords. The stimuli with semantic content but no phonological cues were pictures of objects and animals or their associated sounds. The stimuli with no sublexical phonological cues and no semantic content were colored meaningless scrambled pictures and human humming sounds.

#### Participant instructions

In the speech production conditions, participants were instructed to (a) repeat the auditory words, (b) repeat the auditory pseudowords, (c) name the source of the environmental sounds (e.g., “CAT” in response to “meow”), (d) name the gender of the humming voice (“MALE” or “FEMALE”), (e) read words, (f) read pseudowords, (g) name objects in pictures, and (h) name the dominant color in meaningless pictures of nonobjects. The one-back matching task allowed us to compare the effect of the same stimuli in different tasks because exactly the same stimuli were presented in the eight speech production and eight one-back matching conditions.

### Stimulus selection/creation

Stimulus selection started by generating 128 pictures of easily recognizable animals and objects (e.g., cow, bus, elephant, plate) with one to four syllables (mean = 1.59; *SD* = 0.73). Visual word stimuli were the written names of the 128 objects, with 3 to 12 letters (mean = 5 letters; *SD* = 1.8). Auditory word stimuli were the spoken names of the 128 objects (mean duration = 0.64 s; *SD* = 0.1), recorded by a native speaker of English with a Southern British accent approximating Received Pronunciation. Pseudowords were created using a nonword generator (Duyck et al., 2004), and matched to the real words for bigram frequency, number of orthographic neighbors, and word length. The same male speaker recorded the auditory words and pseudowords.

The non-verbal sounds associated with objects were available and easily recognizable for a quarter (i.e., 32/128) of the (word/picture) stimuli, and taken from the NESSTI sound library (http://www.imaging.org.au/Nessti; Hocking et al., 2013). The duration of the nonverbal sounds needed to be significantly longer (mean length = 1.47 s, *SD* = 0.13) than the duration of the words [*t*_(126)_ = 37.8; *p* < 0.001] because shorter sounds were not recognizable. The auditory baseline stimuli were recorded by both a male and a female voice humming novel pseudowords, and therefore did not carry lexical phonological or semantic content (mean length = 1.04 s, *SD* = 0.43). The male and female voices used to record the baseline stimuli were not used to record the auditory words and pseudowords. Half of these stimuli were matched to the length of the auditory words; the other half, to the length of the nonverbal sounds. The visual baseline stimuli were meaningless object pictures, created by scrambling both global and local features of the original object pictures, then manually editing those pictures to accentuate one of eight colors (brown, blue, orange, red, yellow, pink, purple, or green). We conducted a pilot study with these stimuli with 19 participants, to confirm that they elicited consistent speech production responses.

### Stimulus and task counterbalancing

The 128 object stimuli were divided into four sets of 32 (A, B, C, and D). Set D was always presented as nonverbal sounds. Sets A, B, and C were rotated across pictures, visual words, and auditory words in different participants. All items were therefore novel on first presentation of each stimulus type (for task 1), and the same items were repeated for task 2. Half the participants (13/25) performed all eight speech production tasks first (task 1) followed by all eight one-back matching tasks (task 2). The other half (12/25) performed all eight one-back matching tasks first (task 1) followed by all eight speech production tasks (task 2). Within each task, half the participants (13/25) were presented auditory stimuli first, followed by visual stimuli; and the other half (12/25) were presented visual stimuli first followed by auditory stimuli. The order of the four stimulus types was fully counterbalanced across participants, and full counterbalancing was achieved with 24 of our 25 participants.

Each set of 32 items was split into four blocks of eight stimuli, with one of the eight stimuli repeated in each block to make a total of 9 stimuli per block (eight novel, one repeat). The stimulus repeat only needed to be detected and responded to (with a finger press) in the one-back matching tasks.

### Data acquisition

Functional and anatomical data were collected on a 3T scanner (Trio, Siemens, Erlangen, Germany) using a 12-channel head coil. To minimize movement during acquisition, a careful head fixation procedure was used when positioning each participant's head. This ensured that none of the speech sessions were excluded after checking the realignment parameters. Functional images consisted of a gradient-echo planar imaging (EPI) sequence and 3 × 3 mm in-plane resolution (TR/TE/flip angle = 3080/30 ms/90°, field of view (EFOV) = 192 mm, matrix size = 64 × 64, 44 slices, slice thickness = 2 mm, interslice gap = 1 mm, 62 image volumes per time series, including five “dummies” to allow for T1 equilibration effects). The TR was chosen to maximize whole brain coverage (44 slices) and to ensure that slice acquisition onset was offset-asynchronized with stimulus onset, which allowed for distributed sampling of slice acquisition across the study (Veltman et al., 2002).

For anatomical reference, a high-resolution T1 weighted structural image was acquired after completing the tasks using a three-dimensional Modified Driven Equilibrium Fourier transform (MDEFT) sequence (TR/TE/TI = 7.92/2.48/910 ms, flip angle = 16°, 176 slices, voxel size = 1 × 1 × 1 mm). The total scanning time was approximately 1 h and 20 min per participant, including set-up and the acquisition of the anatomical scan.

### Procedure

Prior to scanning, each participant was trained on all tasks using a separate set of all training stimuli except for the environmental sounds which remained the same throughout both training and experiment. All speaking tasks required the participant to produce a single verbal response after each stimulus presentation by saying the object name, color name, gender, or pseudoword. For the one-back-matching task, participants had to use two fingers of the same hand (12 participants used the right hand, and the other 13 used the left) to press one of two buttons on a fMRI compatible button box to indicate whether the stimulus was the same as the one preceding it (left button for “same,” right button for “different”). This condition did not involve any overt speech but was expected to involve short term memory, supported by “inner” (covert) speech. Participants were also instructed to keep their body and head as still as possible and to keep their eyes open throughout the experiment and attend to a fixation cross on the screen while listening to the auditory stimuli. An eye tracker was used to ensure that participants had their eyes open and paid constant attention throughout the experiment.

Each of the 16 tasks was presented in a separate scan run, all of which were identical in structure.

The script was written with COGENT (http://www.vislab.ucl.ac.uk/cogent.php) and run in Matlab 2010a (Mathsworks, Sherbon, MA, USA). Scanning started with the instructions “Get Ready” written on the in-scanner screen while five dummy scans were collected. This was followed by four blocks of stimuli (nine stimuli per block, 2.52 s inter-stimulus-interval, 16 s fixation between blocks, total run length = 3.2 min). Every stimulus block was preceded by a written instruction slide (e.g., “Repeat”), lasting 3.08 s each, which indicated the start of a new block and reminded participants of the task. Visual stimuli were each displayed for 1.5 s. The pictures subtended an angle of 7.4° (10 cm on screen, 78 cm viewing distance) with a pixel size of 350 × 350, with a screen resolution of 1024 × 768. The visual angle for the written words ranged from 1.47 to 4.41° with the majority of words (with five letters) extending 1.84 to 2.2°.

Auditory stimuli were presented via MRI compatible headphones (MR Confon, Magdeburg, Germany), which filtered ambient in-scanner noise. Volume levels were adjusted for each participant before scanning. Each participant's spoken responses were recorded via a noise-cancelling MRI microphone (FOMRI IIITM Optoacoustics, Or-Yehuda, Israel), and transcribed manually for off-line analysis.

### Data pre-processing

We performed fMRI data preprocessing and statistical analysis in SPM12 (Wellcome Trust Centre for Neuroimaging, London, UK), running on MATLAB 2012a (MathWorks, Natick, MA, USA). Functional volumes were (a) spatially realigned to the first EPI volume and (b) un-warped to compensate for non-linear distortions caused by head movement or magnetic field inhomogeneity. We used the unwarping procedure in preference to including the realignment parameters as linear regressors in the first-level analysis because unwarping accounts for non-linear movement effects by modeling the interaction between movement and any inhomogeneity in the T2^*^ signal. After realignment and unwarping, we checked the realignment parameters to ensure that participants moved less than one voxel (3 mm) within each scanning run.

The anatomical T1 image was (c) co-registered to the mean EPI image which had been generated during the realignment step and then spatially normalized to the Montreal Neurological Institute (MNI) space using the new unified normalization-segmentation tool of SPM12. To spatially normalize all EPI scans to MNI space, (d) we applied the deformation field parameters that were obtained during the normalization of the anatomical T1 image. The original resolution of the different images was maintained during normalization (voxel size 1 × 1 × 1 mm^3^ for structural T1 and 3 × 3 × 3 mm^3^ for EPI images). After the normalization procedure, (e) functional images were spatially smoothed with a 6 mm full-width-half-maximum isotropic Gaussian Kernel to compensate for residual anatomical variability and to permit application of Gaussian random-field theory for statistical inference (Friston et al., 1995).

#### First-level analyses

In the first-level statistical analyses, each pre-processed functional volume was entered into a subject specific, fixed-effect analysis using the general linear model (Friston et al., 1995). All stimulus onset times were modeled as single events, with two regressors per run, one modeling instructions and the other modeling all stimuli of interest (including the repeated and unrepeated items). Stimulus functions were then convolved with a canonical hemodynamic response function. To exclude low-frequency confounds, the data were high-pass filtered using a set of discrete cosine basis functions with a cut-off period of 128 s. The contrasts of interest were generated for each of the 16 conditions of interest (relative to fixation). The results of each individual were inspected to ensure that there were no visible artifacts (edge effects, activation in ventricles, etc.) that might have been caused by within-scan head movements.

### Identifying the effects of interest

At the second level, the 16 contrasts for each participant were entered into a within-subjects one-way ANOVA in SPM12. First we identified areas that were activated for auditory word repetition relative to rest using a statistical threshold of *p* < 0.001 uncorrected. The activated voxels were saved as a single binary image file. Second, we repeated the same second level analysis, but this time we included the binary image file as a region of interest. By excluding any voxels that were not activated for auditory word repetition relative to rest, we ensure that all the effects we report are involved in auditory word repetition. The factorial analysis of the 16 different conditions was implemented at the level of statistical contrasts as described below.

#### Factor 1 (stimulus modality)

Auditory processing areas were identified by the main effect of stimulus modality during the one back matching task (Contrast 1). The statistical contrast compared activation for each of the 4 auditory stimuli to each of the four visual stimuli. To ensure that the observed effects were not driven by a subset of the auditory stimuli (e.g., those with sublexical phonology or semantic content), we used the “inclusive masking” option in SPM to exclude voxels that were not activated at *p* < 0.001 uncorrected in each of the four auditory one back matching tasks relative to fixation. We did not use the speech production conditions in the main effect of auditory processing because this would bias the effects toward auditory speech processing, given that all speech production conditions result in auditory processing of the speaker's voice, irrespective of whether the stimuli are auditory or visual.

#### Factor 2 (sublexical phonological input)

The effect of sublexical phonological input was tested by comparing stimuli with sublexical phonological content (words and pseudowords) to stimuli with no sublexical cues (pictures, environmental sounds, colored patterns, and humming sounds). In Contrast 2, this effect of phonology was computed for auditory one-back matching conditions only to identify areas associated with auditory recognition of speech. The two-way interaction of phonological input with stimulus modality was then computed to confirm whether any phonological effects were specific to the auditory modality.

In Contrast 3, the effect of phonology was computed across both tasks and stimulus modalities to identify activation related to abstract representations of speech sounds or articulatory re-coding. In Contrast 4, the effect of phonology was computed for the one-back matching task only (across stimulus modalities) to identify areas associated with covert articulatory processing which might occur for phonological stimuli during the silent one-back matching task but for all stimuli when speech production was required. When a phonological effect was specific to the one-back matching task (i.e., in Contrast 4), we checked (i) the two way interaction of phonological input and task (one back matching > speech production); and (ii) whether the same regions were activated in the main effect of speech (Contrast 8 below) as this would be consistent with a role for the identified areas in articulatory processing.

In Contrast 5, the effect of phonology was computed for the speech production task only (across stimulus modalities) to identify areas where the motor execution of speech was influenced by sublexical phonological processing. Any such effects were checked with the two-way interaction of phonological input and task (speech production > one back matching).

#### Factor 3 (semantic content)

The effect of semantic input was tested by comparing all stimuli with semantic content (words, pictures, environmental sounds) to all stimuli with no semantic content (pseudowords, colored patterns, and humming sounds). In Contrast 6, this was computed across both tasks and stimulus modalities to identify activation related to accessing semantic associations. In Contrast 7, this was computed for speech production only to identify semantic activation that drove speech production responses. When semantic effects were observed in Contrast 7, we tested whether the effects were significantly enhanced during speech production, by computing the two-way interaction of semantic content with task; and the three way interaction of semantic content with task and stimulus modality.

#### Factor 4 (speech production)

The effect of speech production was tested by comparing all 8 speech production conditions to all eight one-back matching conditions (i.e., Contrast 8). We then separated activation related to the motor execution of speech (orofacial, larynx, and breathing) from activation that was related to auditory processing of the spoken response or domain general processing by looking at which brain areas overlapped with those identified in the main effect of auditory relative to visual input (Contrast 1) or the main effect of all 16 conditions relative to fixation (Contrast 9). Areas associated with motor execution of speech were those that were not activated (*p* > 0.05 uncorrected) in Contrast 1 or during any of the one-back matching tasks that required silent finger press responses (Contrast 9). Areas associated with auditory processing of the spoken response were those that were also activated for auditory relative to visual processing in Contrast 1 as well as speech production relative to one-back matching in Contrast 8.

#### Domain general processing

This was identified where activation was significantly activated in all speech production and all one back matching conditions relative to fixation (Contrast 8). We dissociate areas that were common to Contrasts 8 and 9 (i.e., domain general but enhanced during speech production) from those that were independent of speech production (i.e., Contrast 9 only).

The statistical threshold for all main effects was set at *p* < 0.05 after family wise error correction for multiple comparisons across the whole brain in either height or extent. Within the identified areas, we report interactions if they were significant at *p* < 0.001 uncorrected.

## Results

### In scanner behavior

For technical reasons, button press responses were lost for three participants. Therefore in-scanner behavioral measures were based on all 25 participants for speech production but only 22 participants for the one-back matching tasks. In-scanner accuracy was high (>95%) for all conditions except auditory repetition and reading of pseudowords (88 and 85% respectively) and one-back matching of gender and colors (88 and 95% respectively). The lower accuracy for color and gender arose because some participants attempted to match these stimuli on their visual or auditory forms, rather than their color or pitch. Response times were only available for the one-back matching task and were measured from stimulus onset to response onset. As the time to present each stimulus varied across conditions, we expected the response times to be longer when the stimulus presentation time was longer. For example, in the visual conditions, all visual features are presented simultaneously and then remain on the screen throughout the stimulus duration. In contrast, in the auditory conditions, auditory features emerge over time. Consequently, the response times were slower for the four auditory one-back matching tasks (range across tasks = 880–1125 ms) than the four visual one matching tasks (range across tasks = 648–762 ms). Within the auditory conditions, response times were slower for sound and gender matching (1111 and 1125 ms) than auditory word or pseudoword matching (880 and 959 ms). Within the visual modality, color matching (762 ms) was slower than visual word, pseudoword or picture matching (655, 648, and 683 ms). We think this is because participants were distracted by the shape of the stimulus which changed on each trial irrespective of whether the color was changing.

### fMRI activation results

#### Factor 1: Auditory processing (see blue areas in Figures 2–4)

As expected, activation was significantly greater for auditory than visual stimuli in bilateral superior temporal gyri, including Heschl's gyri and plana temporale. At a statistical threshold of *p* < 0.05 corrected for multiple comparisons, there were 960 voxels in the right auditory cortex and 913 voxels in the left auditory cortex. These areas are associated with auditory processing.

#### Factor 2: phonological inputs (see Table 5A and turquoise areas in Figures 2–4)

There was no effect of phonology in the auditory one back matching task (Contrast 2) that was testing for areas that might be involved in recognizing auditory speech. However, four other phonological effects were dissociated that were all common to auditory and visual inputs. The first two of these were identified in the main effect, across stimuli and tasks (Contrast 3) which was observed in the left superior temporal sulcus (STS) and posterior putamen. We dissociate the function of these areas because in the left STS, activation was additive with a main effect of auditory vs. visual inputs (Z scores for C1 = 5.7 in anterior left STS and more than 8 (i.e., assessed as “effectively infinite” by SPM) in posterior left STS); whereas in the left posterior putamen, the effect was additive with the main effect of speech production (Table 6). Third, during the one-back matching task only (Contrast 3), there was a main effect of phonology in the left pars orbitalis on the junction with the pars triangularis. At the same location, there was an additive effect of speech production (Zscore in C8 = 4.4). Fourth, during the speech production task only (Contrast 5), there was a main effect of phonology in the left ventral premotor cortex and the left anterior putamen. These effects were also additive with the main effect of speech production (see Table 6 for details).

#### Factor 3: semantic content (see Tables 5B,C and pink areas in Figures 2–4)

Three different semantic responses were dissociated. First, a ventral part of the left pars orbitalis was activated by semantic input across stimuli and tasks (Contrast 6). Second, during speech production (Contrast 7) but not one-back matching, the pars orbitalis activation extended more laterally and dorsally, bordering the area associated with phonological inputs during the one back matching task. In addition, semantic inputs during speech production (Contrast 7) increased activation in the left posterior middle temporal gyrus extending into the left angular gyrus and the left hippocampus. Third, there was a three way interaction of semantic content, task, and stimulus modality with auditory semantic inputs (environmental sounds and words) that enhanced activation in left ventral frontal lobe regions (frontal operculum, pars triangularis and the inferior frontal sulcus), and the right cerebellum (laterally in lobule VIIIA and medially in lobule VI).

#### Factor 4: Overt speech production (see Table 6, green areas in Figures 2–6)

**Figure 2:**
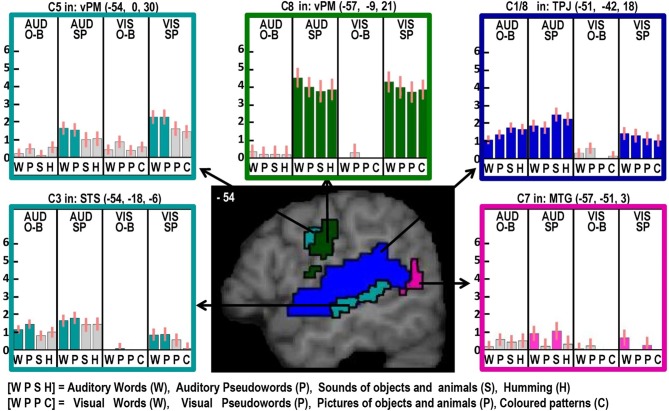
**Illustrations of activations related to auditory processing (in blue), phonological processing (in turquoise), semantic processing (in pink) and the motor execution of speech (in dark green) on a single sagittal brain image at *x* = −54 in MNI space**. Plots show the response for each of the 16 conditions in each of the regions of interest, with the name of the brain region, the x, y, and z MNI co-ordinates of the effect and the Contrast number (e.g., C8) used to identify the effect. The order of the conditions is always the same with abbreviations defined at the bottom of the figure. The conditions of interest for each effect are highlighted in the corresponding color. Red bars on each plot are the 90% confidence intervals generated in SPM. The height of the bar is the mean effect across subjects in arbitrary units, as generated in SPM.

**Figure 3:**
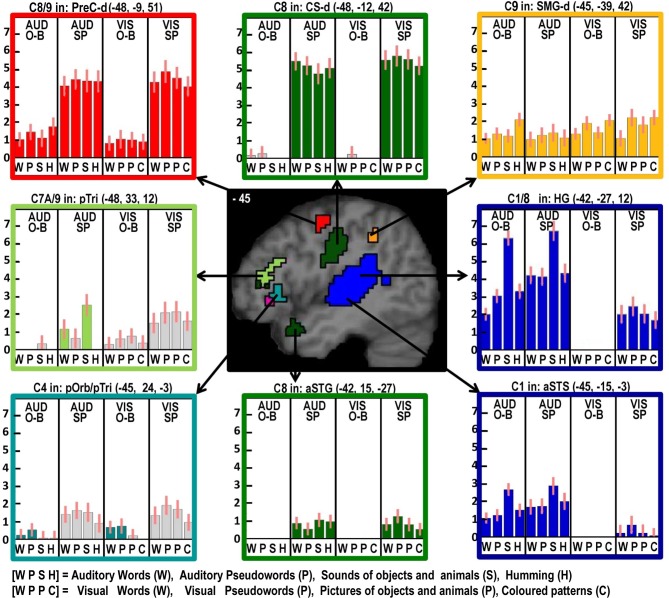
**As in Figure 2 but with effects located at *x* = −45 in MNI space**. Effects colored yellow/orange are those related to domain general processing. Effects in red are those that show an effect of domain general processing (C9) which is enhanced during speech production (C8). The effect in light green was identified for producing speech from auditory semantic stimuli (C7A).

**Figure 4:**
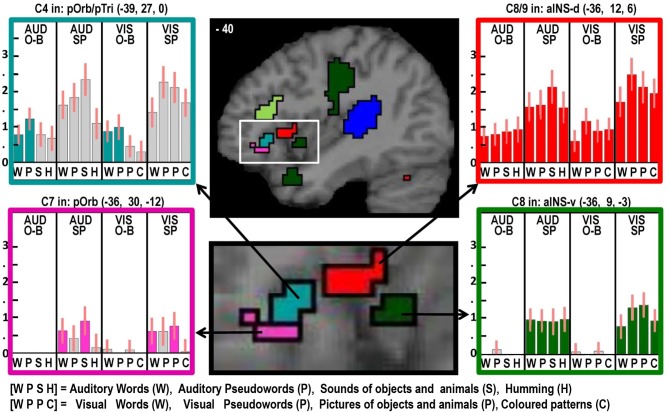
**As in Figures 2, 3 but with effects located at *x* = −40 in MNI space to highlight multiple effects in the left insula and frontal operculum within the white box (brain image above), which is enlarged (brain image below)**.

**Figure 5:**
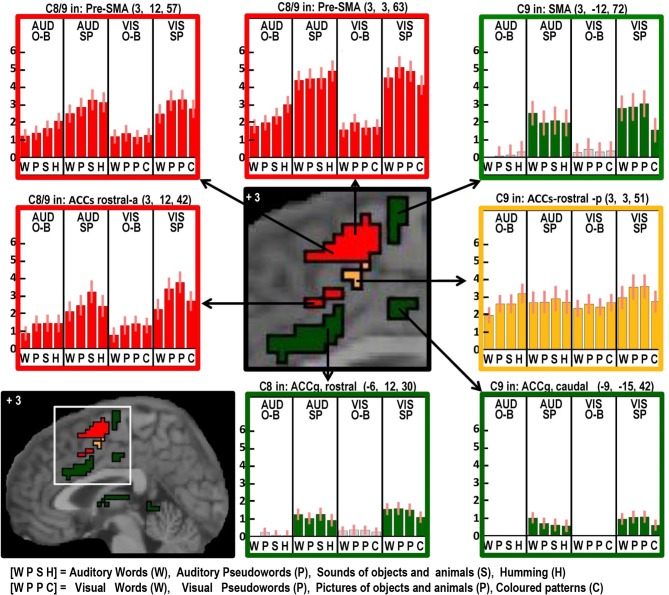
**As in Figures 2–4 but with effects located at *x* = +3 in MNI space to highlight the many different effects in the anterior cingulate and supplementary motor cortex**.

**Figure 6:**
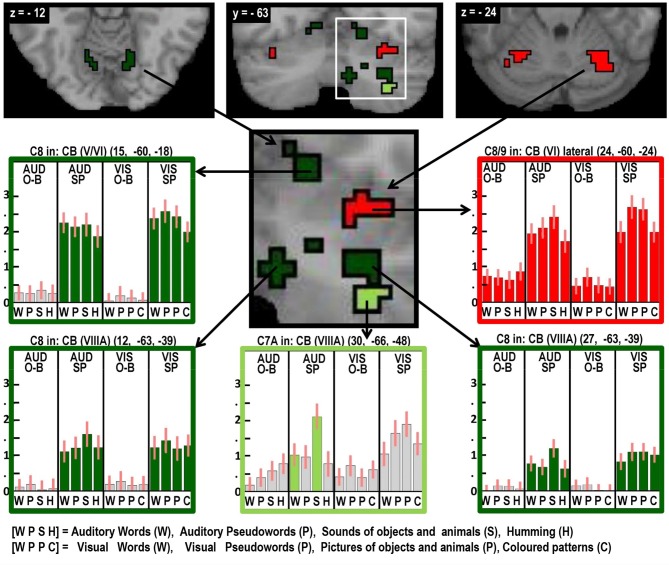
**As in Figures 2–5, highlighting many different auditory repetition effects in the cerebellum**.

Two different sets of speech production responses were dissociated from the main effect of speech production more than one back matching (Contrast 8) after areas associated with domain general processing (Contrast 9) were excluded. First, activation associated with the motor execution of speech was identified bilaterally in the SMA and anterior cingulate gyrus, precentral gyri (including the left ventral premotor cortex activated in C5), many posterior and anterior regions in the insula and putamen, the amygdala, temporal pole, pars triangularis extending into the left pars orbitalis (*Z* = 4.4 at −45, +27, −3) and the cerebellum (green areas in Figures 2–6). Notably, the speech production effects in the left ventral premotor cortex, anterior and posterior putamen and left pars orbitalis were additive with the main effect of phonology reported above and in Table 5A.

**Table 5:** **The effects of phonological vs. semantic inputs. Separate tables indicate the location and significance of activation for: (A) stimuli with phonological vs. non-phonological content; (B) stimuli with semantic vs. non-semantic content; and (C) auditory stimuli with semantic content**.

**PHONOLOGICAL AND SEMANTIC ACTIVATION DETECTED FOR CONTRASTS 3–7 WITH HEMISPHERE (H), MNI CO-ORDINATES (*XYZ*), AND *Z* SCORES FOR EACH TASK**										
**C**	**Location**	**H**	***x***	***y***	***z***	**OB&SP**	**OB**	**SP**	**OB > SP**	**SP > OB**
**(A) PHONOLOGICAL > NON-PHONOLOGICAL INPUT IN AUDITORY AND VISUAL MODALITIES**										
C3	aSTS	L	−54	−18	−6	5.1^*^	3.7^∧^	3.9	ns	ns
C3	pSTS	L	−51	−39	3	4.4^∧^	4.0^∧^	2.6	ns	ns
C3	PUT post	L	−27	−9	−3	5.4^*^	3.1	4.9^*^	ns	ns
C3/5	PUT ant	L	−21	6	3	3.5^∧^	ns	4.7^*^	ns	3.6
C3/5	vPM	L	−54	0	30	4.3^∧^	ns	5.4^*^	ns	3.6
C3/5	vPM	L	−57	6	21	3.2^∧^	ns	4.4^∧^	ns	4.0
C4	pOrb/pTri	L	−45	27	−3	3.8	3.8^∧^	ns	3.0	ns
**(B) SEMANTIC > NON-SEMANTIC INPUT IN AUDITORY AND VISUAL MODALITIES**										
C6	pOrb	L	−33	30	−12	6.6^*^	3.6	6.6	ns	2.6
C7	pOrb	L	−45	30	−6	3.1	ns	4.4^∧^	ns	3.1
C7	pMTG	L	−51	−60	6	4.6^*^	ns	5.8^*^	ns	4.1
C7	pMTG	L	−57	−51	3	3.4	ns	5.8^*^	ns	4.7^*^
C7	ANG	L	−54	−57	18	3.5	ns	4.4^∧^	ns	2.7
C7	Hippocampus	L	−27	−15	−12	4.4^∧^	ns	5.1^*^	ns	2.8
**(C) SEMANTIC > NON-SEMANTIC INPUT DURING AUDITORY PRODUCTION ONLY**										
C7A	FO	L	−30	27	3	ns	ns	4.6^*^	ns	4.5
C7A	pTri	L	−48	33	12	5.5	ns	6.9^*^	ns	4.8
C7A	IFS	L	−39	24	21	5.1	ns	6.2^*^	ns	4.1
C7A	CB (VIIIA)	R	30	−66	−48	ns	ns	5.2^*^	ns	5.2
C7A	CB (VIIIA)	R	27	−66	−39	3.5	ns	4.6^*^	ns	3.4
C7A	CB (VI)	R	9	−72	−21	ns	ns	4.8^*^	ns	4.6

The first column (C) reports the contrast number used in Table 1. The second column gives the anatomical name of the location of the activation using abbreviations explained in Table 4. The third column indicates the hemisphere (H), either left (L), or right (R). The fourth column gives the x, y, and z co-ordinates of the peak activation in MNI space. The last five columns give the Z scores for the effect of interest over task contrast OB&SP, OB only, SP only, OB > SP, and SP > OB. Note that the latter two effects indicate the interaction of task with the effect of interest (phonology or semantics). The main effect of task (over semantic and phonological stimuli) is reported Table 6. Effects that survived family wise error correction for multiple comparisons are marked “^*^” and “^∧^” for height and extent, respectively. Effects that were not significant at p < 0.01 uncorrected are marked ns.

Second, activation associated with auditory processing of the spoken output (i.e., in areas that were also activated for auditory inputs in Contrast 1) was most significant (*p* < 0.05 corrected for multiple comparisons) in dorsal superior temporal gyri. These regions included the left anterior and posterior STS areas associated with the main effect of phonological input (C3; Table 5A). When the significance level was lowered to *p* < 0.001 uncorrected, the main effect of speech production (Contrast 8) was observed in 98% (938/960) of the right hemisphere auditory processing voxels (from Contrast 1) and 88% (801/913) of the left hemisphere auditory processing voxels (from Contrast 1). The absence of an effect of speech production in 12% of the left hemisphere auditory processing voxels suggests that these voxels are not responding to the sound of the spoken response. This apparent discrepancy between the effect of auditory processing of the speakers own voice (in C8) and that of another's voice (C1) will be investigated in a subsequent paper.

#### Domain general processing: (Table 6, red and orange areas in Figures 3–6)

Two different sets of domain general processing areas were dissociated from Contrast 9. Those that were enhanced by speech production (i.e., common to Contrast 8 and 9) were observed bilaterally in the pre-SMA, anterior cingulate sulcus, dorsal precentral gyrus, dorsal anterior insula (around the frontal operculum), and lateral regions of the cerebellum (red in Figures 3–6). Those that were independent of speech production (i.e., Contrast 9 only) were observed in the middle of the anterior cingulate sulcus and a dorsal region of the supramarginal gryus (orange in Figures 3, 5).

**Table 6:** **Brain areas associated with motor execution of speech, domain general processing, or both**.

**MOTOR EXECUTION OF SPEECH AND DOMAIN GENERAL PROCESSING**						
**MOTOR EXECUTION OF SPEECH (CONTRAST 8)**						
**Location**		**H**	***x***	***y***	***z***	**Zsc**
SMA		R	3	−12	72	6.5
ACCg	rostral	L	−6	12	30	7.0
		R	6	15	30	7.6
	caudal	L	−9	−15	42	7.7
		R	6	−12	39	6.2
PrCg/CS		L	−48	−12	42	Inf
		R	48	−9	39	Inf
		L	−18	−30	54	Inf
		R	18	−27	60	Inf
PrCg-v		L	−57	−9	21	Inf
		R	60	−6	18	Inf
vPM		L	−57	3	21	6.6
		R	57	3	21	Inf
pINS-d		L	−33	−12	18	Inf
		R	33	−12	18	Inf
aINS-d		L	−33	6	12	6.3
		R	36	9	9	6.8
aINS-v		L	−30	9	−9	7.6
		L	−36	9	−3	6.9
		L	−30	−6	−9	Inf
		R	30	12	−9	7.7
		R	33	9	0	7.1
		R	42	9	−3	6.5
		R	30	−9	−6	Inf
PUT post		L	−27	−12	−6	Inf
		R	−27	−12	−6	Inf
PUT ant		L	−21	6	3	Inf
		R	21	6	3	Inf
Amygdala		L	−27	−3	−15	Inf
		R	30	−3	−15	Inf
TP/aSTG		L	−42	15	−27	7.2
		L	−51	12	−18	5.8
		R	39	15	−30	7.5
		R	48	12	−24	7.0
**MOTOR EXECUTION OF SPEECH (CONTRAST 8)**						
pTri		L	−48	39	9	6.5
		L	−48	30	6	5.7
		R	51	39	9	7.6
		R	45	30	3	7.0
CB (V/VI)		L	−12	−66	−12	Inf
Paravermal		R	15	−60	−18	Inf
		R	15	−72	−15	Inf
CB (VIIIA)		L	−24	−69	−42	5.3
Paravermal		R	12	−63	−39	7.8
		R	9	−66	−36	7.6
**DOMAIN GENERAL PROCESSING AREAS**						
*where activation is enhanced during*						
**OVERT SPEECH (Z SCORES FROM CONTRAST 9)**						
Pre-SMA			3	3	63	Inf
			3	12	57	6.3
ACCs		L	−6	12	39	6.1
		R	9	15	36	6.8
		R	3	12	42	5.9
PrCg-d		L	−48	−9	51	Inf
		R	51	0	45	Inf
PM-v		L	−60	3	24	Inf
aINS-d/FO		L	−36	12	6	5.4
		L	−39	27	0	5.1
		R	36	18	6	5.3
		R	39	24	6	5.3
CB (VI)		L	−27	−60	−24	Inf
Lateral		L	−36	−60	−24	6.6
		R	24	−60	−24	Inf
**DOMAIN GENERAL PROCESSING (CONTRAST 9)**						
SMA		L	−6	0	54	Inf
		R	3	3	51	Inf
SMG-d		L	−45	−39	42	Inf

*Columns 1–3 in each section correspond to columns 2–4 in Table 5. Column 4 reports the Z score (Zsc) for the main effect of speech production (Contrast 8) or domain general processing (Contrast 9). Activation in auditory processing areas (from Contrast 9) is excluded (see results section for details)*.

In summary, the fMRI results replicated many previous findings (Table 2A) but also revealed many novel effects (Table 2B) which we now discuss.

## Discussion

In this paper, we have identified multiple regions activated during auditory repetition of words compared to resting with eyes open (i.e., fixation), then used multiple different contrasts, within-subject, to try to assign functional roles to those regions. Our results extend, refine, and in some cases undermine prior predictions about what regions support auditory word repetition, and what those regions actually do. In what follows, we discuss the results in the context of those prior expectations, focusing on phonological processing, semantic processing, and motor execution of speech during auditory word repetition.

### Phonological effects

We found no regions that were specifically responsive to the auditory processing of speech (i.e., no effect of phonological inputs on auditory relative to visual stimuli in C2). However, we did find four effects of phonological input that were common to auditory and visual stimuli. Across tasks (C3), phonological inputs increased activation in the left STS and the left posterior putamen. In addition, during speech production, phonological inputs increased activation in the left ventral premotor cortex and the left anterior putamen (C5); and during one-back matching, phonological inputs increased activation in the left pars orbitalis (C4). Below, the role that each of these regions might play in phonology is discussed.

#### Left STS

Here we observed a main effect of phonological inputs (C3) that was additive with a main effect of auditory vs. visual processing (C1), see lower left of Figure 2. We predicted such a response in left posterior STS but not left anterior STS. However, the effect we observed anteriorly (at −54, −18, −6) is consistent with a previous study that reported an anterior STS region (at −50, −22, −6) with an additive effect of (i) auditory vs. visual stimuli and (ii) reading and repetition of speech relative to non-speech (Price et al., 2003). What might this auditory processing area be doing during reading? Examination of the plot in the lower left corner of Figure 2 shows that, although the effect of phonology on auditory stimuli was consistent for both tasks, the response to visual phonological inputs was primarily observed during speech production rather than one-back matching. This could either arise because (A) reading aloud increases access to auditory representations (phonology) more than naming pictures (Glaser and Glaser, 1989); or (B) participants enhance auditory processing of their spoken response during reading relative to naming (even though the auditory input from the spoken response is matched in the reading words and naming picture conditions). We exclude the latter explanation (B), because the common effect of auditory and visual phonology reported in Price et al. (2003) was observed in the context of silent speech production (moving lips without generating any sound) which eliminated auditory processing of the spoken response. We therefore focus on explanation (A), i.e., the left aSTS response reflects access to auditory representations of speech that are readily accessed during reading. Indeed, many previous studies have reported extensive STS activation in the context of audio-visual integration (Calvert et al., 1999; Noppeney et al., 2008; Werner and Noppeney, 2010).

#### Left posterior putamen

Here we observed an additive effect of phonological input (C3) and speech production (C8). The response in this region, from the same dataset, has been discussed at length in Oberhuber et al. (2013), which investigated differential activation for reading words and pseudowords and found higher left posterior putamen activation for reading and repeating words than reading or repeating pseudowords (see Figure 2 in Oberhuber et al., 2013). This was interpreted in light of other studies that have associated the posterior putamen with “well learnt movements” (Menon et al., 2000; Tricomi et al., 2009). As articulation is matched in the reading and picture naming conditions, increased left posterior putamen activation for reading must reflect pre-articulatory processing, particularly since left posterior putamen activation was also detected for phonological inputs during the one-back task that did not require overt articulation. We therefore speculate that the effect of phonological inputs on left posterior putamen responses reflected activation related to articulatory planning.

#### Left ventral premotor cortex

Here we observed an effect of phonological input during speech production (C5) and an additive effect of speech production (C8). Phonological inputs may increase the demands on overt articulation because they provide multiple sublexical phonological cues that need to be integrated (sequenced) into a lexical motor plan. This would be consistent with left ventral premotor cortex playing a role in articulatory sequencing. Contrary to our expectations, we found no evidence for covert articulatory processing in this ventral premotor area during the one-back matching task.

#### Left anterior putamen

Here we observed a pattern of response that was similar to that observed in the left ventral premotor cortex: i.e., an effect of phonological input during speech production (C5) and an additive effect of speech production (C8). In addition, using the same dataset, we have previously reported that the left anterior putamen was more responsive during pseudoword reading than word reading (see Figure 2 in Oberhuber et al., 2013). We interpreted this effect as in keeping with prior studies that have associated with left anterior putamen with “the initiation of novel sequences of movements” (Okuma and Yanagisawa, 2008; Aramaki et al., 2011; Wymbs et al., 2012) as opposed to well-known movements in posterior putamen. Keeping with this conclusion provides an interpretation of left anterior putamen activation that is similar to that of the left ventral premotor cortex; i.e., both are involved in sequencing the articulation of sublexical phonological codes.

#### Left pars orbitalis

Here, we observed an effect of phonological input during one-back matching (C4) with an additive effect of speech production (C8). Neither of these effects was expected in the left pars orbitalis, which is more commonly associated with semantic processing (Dapretto and Bookheimer, 1999; Poldrack et al., 1999; Devlin et al., 2003; Gough et al., 2005; Vigneau et al., 2006; Mechelli et al., 2007; de Zubicaray and McMahon, 2009). Nevertheless, there are other studies that have reported left pars orbitalis is sensitive to articulatory complexity during non-semantic pseudoword production (Park et al., 2011) and for reading pseudowords relative to words (Hagoort et al., 1999). Therefore, our study is not unique in highlighting a non-semantic articulatory response in this region.

We controlled for articulatory complexity in our speech production conditions because speech outputs were the same object and animal names in auditory repetition, reading aloud and object naming. Interestingly in this context, we did not see differential activation in the left pars orbitalis for phonological and non-phonological inputs during speech production. Plausibly, the effect of phonological inputs that we observed in the left pars orbitalis during the one back matching task might reflect covert articulatory processing that occurs automatically when stimuli have strong articulatory associations. Future studies should therefore consider the possible involvement of phonological processing when interpreting left pars orbitalis activation. For example, in Leff et al. (2008), we observed left pars orbitalis activation at MNI co-ordinates [−48, 28, −6] for listening to speech relative to reversed speech, and interpreted this effect as reflecting semantic processing. In light of the current study, increased left pars orbitalis to speech may have reflected covert articulatory processing.

To summarize this section, we have dissociated four different phonological effects. All were observed in the left hemisphere, with one in an auditory processing area (STS) and three in speech production areas: (i) during speech production only (left ventral premotor cortex and left anterior insula), which we associate with sequencing sublexical articulatory plans; (ii) during one-back matching only (left pars orbitalis), which suggests covert articulatory processing that is equally involved for phonological and non-phonological stimuli during overt speech production; or (iii) during both tasks (left posterior putamen), which is consistent with increases in both covert and overt articulatory activity.

### Semantic processing

Despite finding that part of the pars orbitalis responded, unexpectedly, to speech production and sublexical phonological inputs during the one back matching task, we found a different part of the left pars orbitalis for the main effect of semantic content (C7). This is consistent with the prior association of the left pars orbitalis with semantic processing mentioned above. The left hand plots in Figure 4 illustrate the strikingly different responses to two distinct but neighboring parts of the left pars orbitalis, with the semantic area (in pink) lying ventrally to the phonological area (in turquoise).

Semantic content also increased activation in the left posterior middle temporal gyrus, extending into the ventral part of the angular gyrus, with a separate peak in the left hippocampal gyrus. These regions were expected during all semantic conditions but were only detected during the speech production conditions (Table 5B), suggesting that semantic associations were weak, or not engaged, during the one back matching. On the other hand, the demonstration that posterior left temporo-parietal areas were involved in auditory word repetition, and other semantic speech production tasks, provides a clear illustration that semantic processing is activated during auditory word repetition, even if it is not theoretically needed.

Likewise, our data suggest evidence that auditory word repetition increases the demands on phonological retrieval mechanisms in left lateralized frontal and right lateralized cerebellar regions that were activated during speech production in response to: (a) semantic relative to non-semantic inputs in the auditory modality; and (b) all conditions relative to fixation, in the visual modality. Activation was therefore least when speech was generated from auditory pseudowords or auditory hums (that have no lexical or semantic representations); see the left middle plot in Figure 3 and lower middle plot in Figure 6. This pattern of response is interesting because auditory words could theoretically be repeated like auditory pseudowords. The fact that auditory word repetition activated areas strongly involved in naming environmental sounds suggests that either the extra processing makes the task easier or it occurs automatically, whatever its actual benefits.

### Motor execution of speech

Turning now to the effects of speech production that were not influenced by semantic or phonological differences in the stimulus input, our experimental design was, for what we think is the first time, able to segregate activation related to the motor execution of speech from that involved in domain general processing and auditory processing of the spoken output.

Areas associated with the motor execution of speech included all those that were predicted, with the exception of the thalamus. In addition, we observed activation in the SMA, anterior cingulate, pars triangularis, and an extensive region around the ventral insula and ventral putamen that included the claustrum and spread into the amygdala and temporal pole. We have replicated the effects of speech production in the temporal pole in another study and this will be discussed in our future communications. Here we focus on the effects in the SMA, anterior cingulate, cerebellum, and anterior insula. Those in the SMA and anterior cingulate were not predicted to be specific to speech output because they have been reported to respond during numerous studies of movement with other effectors such as the hand. Indeed, we found other parts of the cingulate and the pre-SMA were involved in both our speaking and finger-press tasks. Thus our study highlights the many different areas in the medial frontal cortex that are all involved in speech production but differ in their involvement in other tasks. Figure 5 illustrates the relative location of these areas: those in red and orange responded during one back matching and speech production whereas those in green did not respond during one back matching—so appear to be primarily involved in the motor execution of speech. Likewise, Figure 6 illustrates three different types of functional responses in the cerebellum with medial (paravermal) areas being most responsive during speech production and more lateral areas showing sensitivity to retrieval demands (lower middle plot) and one back matching (top right plot). Future connectivity studies could investigate how the different cerebellar regions shown in Figure 6 interact with the medial frontal regions shown in Figure 5.

Finally, we highlight an interesting dissociation in the left anterior insula. Previous studies have associated this area with either covert speech production (Koelsch et al., 2009), or overt speech production (Ackermann and Riecker, 2004; Shuster and Lemieux, 2005; Bohland and Guenther, 2006). Here we dissociate two different areas involved in speech production, see right panel of Figure 4. A dorsal area close to the frontal operculum that is activated during silent and overt speech production conditions (shown in red) and a more ventral area that is specific to overt speech production (shown in green).

### Implications for theories of auditory word repetition

Our results illustrate the complexity of response profiles in many regions involved in auditory word repetition, and distinguish sub-regions within some of those regions that have not been identified before. For example, though we did find evidence consistent with the conventional view that the left pars orbitalis plays a semantic role, we also found more phonological activity in a different part of the same region. Similarly, we were able to dissociate three different regions in the cerebellum, and two different regions within the left anterior insula—all of which are implicated in the motor execution of speech, but each of which responds differently in different tasks. These results suggest that there are multiple, overlapping but at least partially independent circuits involved in auditory word repetition—circuits which are not addressed at all in contemporary models of the process (e.g., Ueno et al., 2011; Ueno and Lambon Ralph, 2013).

## Conclusions

Auditory word repetition is a complex process, supported by a large network of widely distributed brain regions, many of which appear to have their own complex, task-dependent response profiles. As with many other language functions, our analyses of auditory word repetition are hampered first and foremost by that complexity: there are so many regions involved, with such complex response profiles, in such a potentially wide array of different functional circuits, that we cannot hope to explain it all here. Instead, we have sought to show how a multi-factorial, within-subjects design can be used to begin to dissect this complex network, and to illustrate some of the key lessons that can be learned—lessons which go beyond any simple tabulation of those regions that do or do not appear to be implicated by auditory repetition, and which were, or were not, expected to appear in that list (Table 2).

Perhaps the most important lesson of all here is that conventional models of auditory repetition, be they founded on the dual route hypothesis (e.g., Saur et al., 2008; Ueno et al., 2011), or even on our own analysis of many hundreds of previous imaging experiments (see Figure 1), are simply not detailed enough to account for the data we increasingly observe as our imaging methods improve. For example, we show here that there are several different types of phonological response that cannot be explained as a single concept. We have also shown how areas associated with semantic processing and phonological retrieval respond during auditory word repetition, even though they are theoretically not required. For anatomical models of auditory repetition, and language *per se*, we have shown that there are two different regions in the left pars orbitalis, one that responds to semantic content and one that responds to articulatory processing. At the speech output level, we have dissociated multiple different areas and responses that all need further investigation. Nevertheless, some of the results (e.g., those in the left anterior insula) allow us to reconcile previously conflicting reports.

We hope that these and other results will motivate future experiments that investigate, validate and interpret the vast array of neural responses that support even the simplest of language tasks. We also hope that our single experimental paradigm will be useful dissociating language responses at the individual level, particularly in the clinical setting.

## Author contributions

Cathy J. Price, David W. Green, ‘Ōiwi Parker Jones. were responsible for the study design. Thomas M. H. Hope created the paradigm and supervised the data acquisition. Mohamed L. Seghier supervised the data analysis. All authors contributed to and approved the final manuscript.

### Conflict of interest statement

The authors declare that the research was conducted in the absence of any commercial or financial relationships that could be construed as a potential conflict of interest.
